# A Novel Electrochemical Sensor for Detection of Nicotine in Tobacco Products Based on Graphene Oxide Nanosheets Conjugated with (1,2-Naphthoquinone-4-Sulphonic Acid) Modified Glassy Carbon Electrode

**DOI:** 10.3390/nano12142354

**Published:** 2022-07-09

**Authors:** M. Abd-Elsabour, Hesham M. Alsoghier, Abdulrahman G. Alhamzani, Mortaga M. Abou-Krisha, Tarek A. Yousef, Hytham F. Assaf

**Affiliations:** 1 Chemistry Department, Faculty of Science, South Valley University, Qena 83523, Egypt; m.sabour28@sci.svu.edu.eg (M.A.-E.); hgmohmed@gmail.com (H.M.A.); 2 Chemistry Department, College of Science, Imam Mohammad Ibn Saud Islamic University (IMSIU), Riyadh 11623, Saudi Arabia; agalhamzani@imamu.edu.sa (A.G.A.); tayousef@imamu.edu.sa (T.A.Y.); 3 Mansoura Laboratory, Department of Toxic and Narcotic Drug, Forensic Medicine, Medicolegal Organization, Ministry of Justice, Mansoura 35511, Egypt

**Keywords:** adapted glassy carbon sensor, cigarette, differential pulse voltammetry, graphene oxide nanosheets synthesis, nicotine

## Abstract

A simple electrochemical sensor for nicotine (NIC) detection was performed. The sensor based on a glassy carbon electrode (GCE) was modified by (1,2-naphthoquinone-4-sulphonic acid)(Nq) decorated by graphene oxide (GO) nanocomposite. The synthesized (GO) nanosheets were characterized using X-ray diffraction (XRD), Raman spectroscopy, scanning electron microscope (SEM), transmission electron microscope (TEM), FT-IR, and UV-Visible Spectroscopy. The insertion of Nq with GO nanosheets on the surface of GCE displayed high electrocatalytic activity towards NIC compared to the bare GCE. NIC determination was performed under the optimum conditions using 0.10 M of Na_2_SO_4_ as a supporting electrolyte with pH 8.0 at a scan rate of 100 mV/s using both cyclic voltammetry (CV) and differential pulse voltammetry (DPV). This electrochemical sensor showed an excellent result for NIC detection. The oxidation peak current increased linearly with a 6.5–245 µM of NIC with R^2^ = 0.9999. The limit of detection was 12.7 nM. The fabricated electrode provided satisfactory stability, reproducibility, and selectivity for NIC oxidation. The reliable GO/Nq/GCE sensor was successfully applied for detecting NIC in the tobacco product and a urine sample.

## 1. Introduction

Nicotine(S)3-(1-methylpyrrolidin-2-yl) pyridine (NIC) is one of the fatal toxicities of purine alkaloids which represents about 2–8% of the tobacco plants (Nicotiana genus such as N. Tabacum, N. Rustica, and N. Glauca) [1,2,3,4,5]. It is an oily, hygroscopic, colorless pale yellow liquid that quickly dissolves in water at room temperature [6]. Additionally, NIC is rapidly absorbed in humans and directly attacks the nervous system. The frequent intake of NIC creates many potentially harmful diseases and effects on human health, such as increased blood pressure and heartbeat, reduced healing rates, and vascular disease [7,8,9]. Moreover, it might lead to lung, nose, kidney, stomach, bladder, and colon cancer [10,11]. However, it can treat Alzheimer’s and Parkinson’s diseases [12,13].

Hence, great attention has been paid to the determination of NIC, especially in medicine, toxicology, and the tobacco industry. To date, several analytical techniques have been reported for the measurement of NIC, including high-performance liquid chromatography [14], gas chromatography [15], spectrophotometry [16], spectrofluorimetry [17], radioimmunoassay [18], amperometric assay [19], and capillary electrophoresis [4]. Unfortunately, most of these methods possess numerous disadvantages, such as being expensive and time-consuming and requiring several chemical treatment steps and many organic solvents [9,20,21]. Moreover, limited biosensors have been used to analyze NIC due to the high cost of enzymes [22,23].

On the other hand, electrochemical techniques are more convenient for detecting NIC due to their practical advantages, such as low-cost instruments, high sensitivity, good selectivity, simple operation, and rapid measurement time [8,24]. The voltammetric determination of NIC at bare electrodes is hindered by slow electrode kinetics. This is not only the electro-oxidation or reduction reaction of NIC occurring at highly positive or negative redox potentials leading to poorly reproducible results. Chemically modified electrodes are the key to enhance the electrocatalytic ability toward NIC determination due to their higher sensitivity and selectivity than typical electrodes [25].

(1,2-naphthoquinone-4-sulphonic acid) is an organic layer on the surface of the electrode in a rich active group, where there are higher chances of adsorption with the analyte. This leads to enhance the conductivity and increasing the effective surface area of the glassy carbon electrode (GCE).

Additionally, graphene oxide (GO) nanosheets have attracted much attention and are considered an excellent candidate for electrode surface modification, high specific surface area, remarkable electrical conductivity, enhanced electrocatalytic, and high mechanical properties [26,27,28]. The unique conjugation between a film of Nq with GO nanosheets on the surface of the GCE can improve the detecting efficiency, stability, reproducibility, and electrocatalytic properties of electrodes [29,30,31].

Our work aims to benefit from the conjugation between Nq layer with GO nanosheets on the surface of a glassy carbon electrode to determine the NIC for the first time. As far as we know, there are no papers of study of the electrochemical behaviors of nicotine at the GO/Nq/GCE. The mixed modification facilitates enhancing the quality of the glassy carbon electrode. This method was applied to determine Nicotine(S)3-(1-methylpyrrolidin-2-yl) pyridine, NIC. In sequence, actual samples of tobacco products were studied in an aqueous media, and satisfying results were obtained with the proposed sensor.

## 2. Experimental

### 2.1. Materials and Reagents

Graphene powder (<20 μm) and 1,2-naphthoquinone-4-sulphonic acid were purchased from Sigma Aldrich. Aldrich’s stock solution of 6.2 mM NIC (C_10_H_14_N_2_, 98%) was freshly prepared using deionized water and kept in the dark at 4 °C. Before use in the experiments, the stock solution was diluted using sodium sulphate (Na_2_SO_4_, 0.10 M) purchased from Sigma Aldrich as a supporting electrolyte. All other chemical reagents were of analytical grade and used as received without any further purification.

### 2.2. Apparatus

The voltammetric experimental conditions have been described in detail in previous literature [32]. Briefly, EG&G Princeton applied research potentiostat/galvanostat model 263 (USA) linked with a PC using 352 corrosion software [32,33]. A conventional three-electrode cell consisted of a GO/Nq/GCE as the working electrode, a saturated Ag/AgCl reference electrode, and an auxiliary electrode made of a bright platinum wire. A pH meter (Euteoh-India) was employed for the pH measurements. The synthesized GO was characterized by powder X-ray diffraction (XRD, X’Pert3 Powder, PA Analytical, Netherlands), Raman spectroscopy, a field-emission scanning electron microscope (SEM, QUANTA FEG250, Tokyo, Japan), transmission electron microscope (TEM, JEOL 2100 HRTEM 200V, Tokyo, Japan), FT-IR and UV-Visible spectroscopy (Perkin Elmer, Waltham, WA, USA). In this study, all measurements were carried out at room temperature.

### 2.3. Synthesis of GO

**Scheme 1:**
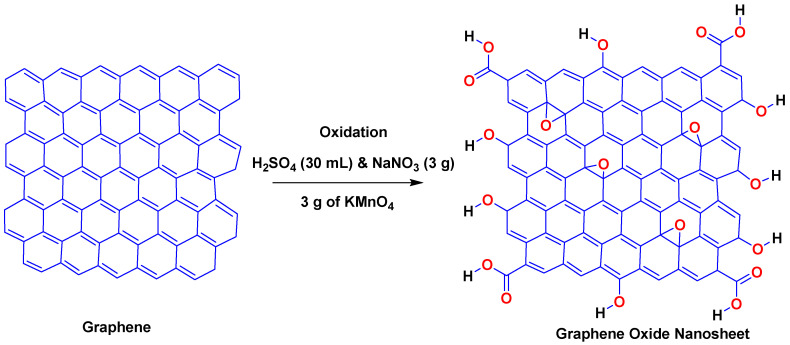
Synthesis of GO using modified Hummers’ method.

Synthesis of GO nanosheets were prepared according to the modified Hummers’ method [34,35,36]. Specifically, in an ice-water bath, 1.0 g of natural graphene powder was mixed with the concentrated H_2_SO_4_(30 mL) and NaNO_3_(3g). Then, 3.0 g of KMnO_4_ was slowly added to keep the oxidation reaction temperature between 10 and 25 °C (Scheme 1). Subsequently, 100 mL of deionized water and 60 mL of 35% H_2_O_2_ were carefully added to reduce residual KMnO_4_ and MnO_2_. Afterward, the mixture was stirred for 30 min and then centrifuged. Finally, the product was filtered and dried in the oven at 50 °C overnight. According to the literature, GO nanosheets were synthesized from the resulting graphene oxide by magnetic stirring and heating [37,38]. Briefly, 0.50 g of dried graphene oxide nanocomposite powder was stirred with 50 mL of deionized water at 70 °C using magnetic stirring at 400 r.p.m for 12 h [38,39,40].

### 2.4. GO/Nq/GCE Fabrication

Before modification, a GCE (1 mm diameter) was sequentially polished with alumina powder (3 µm) to a mirror finish surface and ultrasonically cleaned with water: ethanol for 2 min and rinsed with deionized water. The modification of GCE by a layer of Nq can be obtained by electrodeposition on the cleaned surface of GCE. This was performed by dipping GCE in 0.10 M PBS containing 3.33 mM of Nq monomer and was conditioned by cyclic sweeping from −1.25 to 1.3 V vs. Ag/AgCl for 15 cycles (Section 3.2) [41]. The Nq/GCE was carefully cleaned with deionized water and dried in the air. On the other hand, the Nq/GCE was decorated by prepared GO nanosheets by dissolving the GO nanosheets in deionized water to form a suspended solution subjected to an ultrasonic device for 30 min to form a homogenous suspended solution. Then, 5.0 µL of the homogenous suspended solution (2.0 mg/mL) was cast on the surface of Nq/GCE to form GO/Nq/GCE. Finally, the GO/Nq/GCE was carefully cleaned with deionized water and dried in air. To calculate the activate surface of the different electrodes, the cyclic voltammetry of 5.0 mM of K_3_Fe(CN)_6_as the redox at the surface of bare GCE, Nq/GCE, andGO/Nq/GCE at different scan rates (from 50 to 300 mV/s) were recorded. There is a direct correlation between peak current and scan rate. By increasing the scan rate, the peak current increased for each electrode. By deriving the relationship between Ip and the square root of scan rate for different electrodes. Straight lines were obtained. Moreover, by compensation of the slope in the following Randles–Sevcik equation used [41].

**Ip = 0.446 nFAC(nFDν/RT)^1/2^** (1)

where R is the universal gas constant (J/mol.K), n is the number of electrons, C is the concentration of the electroactive species (mM), F is the Faraday constant (C/mol), ν is the scan rate (mV/s), T is the temperature in Kelvin, Ip is the peak current, D isthe diffusion coefficient (cm^2^/s) and A(cm^2^) is the electroactive surface area of the different types of electrodes. The accurate value of D is 5.6 × 10^−6^ cm^2^/s [42].So the values of active surface area(A cm ^2^) were estimated for bare GCE, Nq/GCE, andGO/Nq/GCE as 0.0126 cm^2^, 0.033 cm^2^,and0.0425 cm^2^, respectively. So GO/Nq/GCE provides superior conductivity to other electrodes according to the active surface area data.

### 2.5. Sample Preparation

For the cigarette samples analysis, leaves were boiled for 5 min in 50 mL of deionized water. The mixture was later cooled and filtered to remove suspended impurities, followed by centrifugation. Decanting the supernatant centrifuged solution resulted in a clean dark brown colored solution. A human urine sample was provided by a project team member and diluted 100 times with 0.10 M Na_2_SO_4_ at pH 8.0.

## 3. Results and Discussion

### 3.1. Characterization of the Synthesized GO

**Figure 1:**
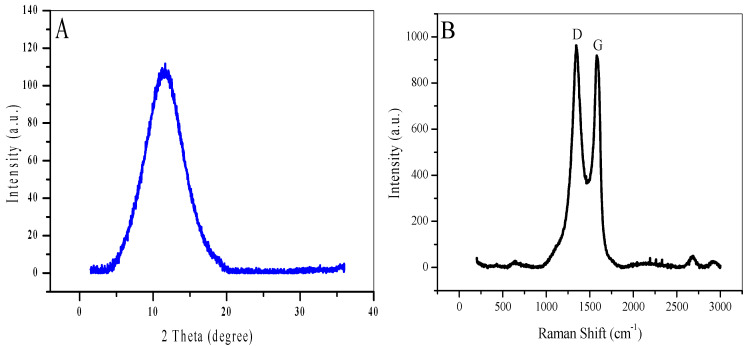
(**A**) XRD and (**B**) Raman spectra of the synthesized GO.

Figure 1A displays the XRD patterns of the GO, which demonstrates that GO was successfully synthesized. Thus, the GO exhibits a broad diffraction peak at 2*θ* of 11.58° due to the interlayer distance of the GO [34]. The Raman spectrum of the synthesized GO has been shown in Figure 1B to confirm the structural properties of GO. Two peaks were observed at 1345 cm^−1^ (D band) and 1581 cm^−1^ a (G band). The D (related to structural defects and amorphous carbon) to G (associated with the stretching of in-plane sp2carbons) intensity ratio (I_D_/I_G_) was 1.05. The Raman spectrum of the GO is consistent with previous reports [42,43].

**Figure 2:**
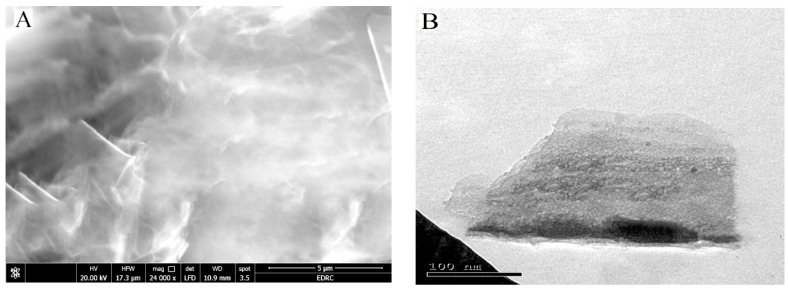
(**A**) SEM and (**B**) TEM images of the synthesized GO nanosheets.

On the other hand, the SEM image of the GO is depicted in Figure 2A to examine its structural morphology. As expected, a typical wrinkled, thin sheet-like morphology is shown for the surface of GO. For further support, the features of morphology and structure of the GO can be observed by high-resolution TEM. Figure 2B clearly shows the single-layer sheets containing flake-like wrinkles and rough surfaces. This microstructure gives the stability of GO nanosheets and prevents the collapse back into graphene structure, which provides a high surface area [44].

**Figure 3:**
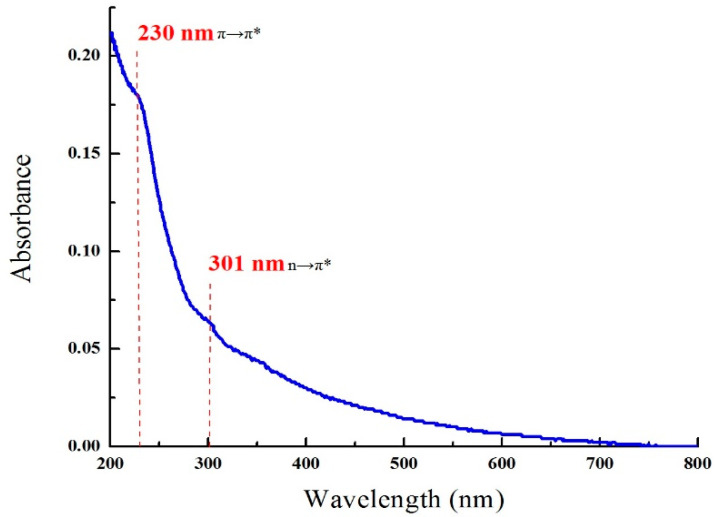
UV-Visible spectrum curve of the aqueous solution of GO nanosheets (0.0023g GO in 4.5 mL distilled H_2_O).

Figure 3 shows the UV–Visible absorption spectrum of the aqueous solution of GO nanosheets. The observed characteristic peak (230 nm) and shoulder (301 nm) are assigned to π→π* transition of aromatic C=C matrix of GO and *n*→π* transition of the oxygen lone pair of electron of the carbonyl group (C=O) in GO, respectively [38,40].

**Figure 4:**
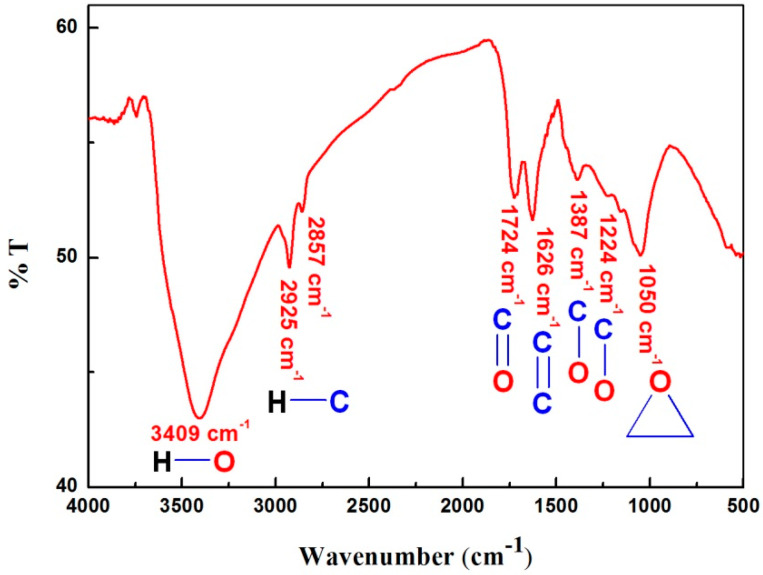
FT-IR spectrum curve of GO in KBr disc.

FT-IR spectroscopy is vital for describing functional moieties involved on GO’s surface. Figure 4 shows the site of absorption bands of functional groups in GO. The existence of broad stretching peaks at 3409 cm^−1^, 2925 cm^−1^, and 2857 cm^−1^ are stretching asymmetric and symmetric a hydroxyl (O–H) and C–H groups of GO matrix [38,39,40,45], respectively. Moreover, the C=O stretching peak of the carboxylic (COOH) group at 1724 cm^−1^, stretching band at 1626 cm^−1^ of aromatic C=C matrix of GO, and phenolic C–O deformation, stretching, and epoxy C–O–C stretching were recognized at 1387, 1224 and 1050 cm^−1^, respectively [38,39,40,46,47].

### 3.2. Electrochemical Behaviorof Nq

**Figure 5:**
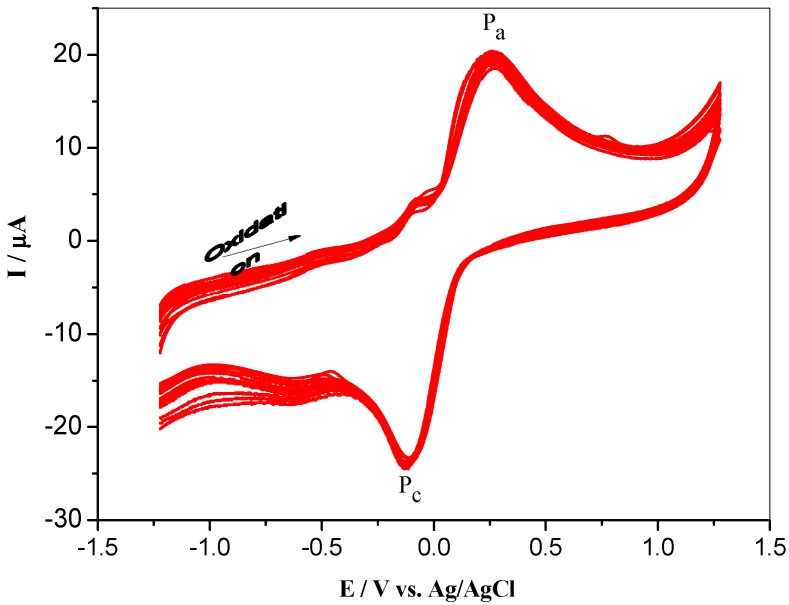
CVs of 3.33 mM of Nq in 0.10 M of PBS (pH 7.0) at the GCE with a scan rate of 100 mV/s.

**Figure 6:**
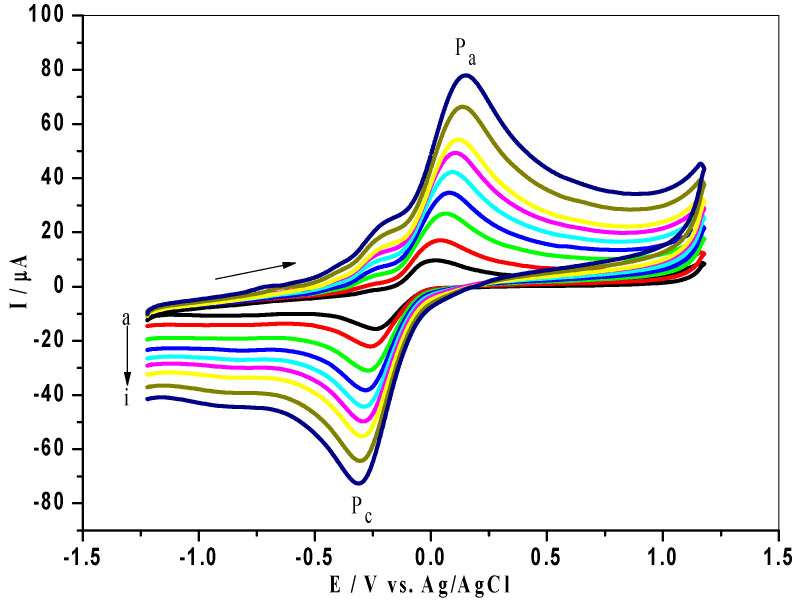
CVs of the Nq/GCE in 0.10 M of PBS (pH 7.0) at various scan rates (a to i: 25, 50, 100, 150, 200, 250, 300, 400, and 500 mV/s).

Figure 5 depicts the cyclic voltammograms (CVs) of 3.33 mM of Nq in 0.10 M PBS (pH 7.0) on the surface of GCE at a scan rate of 100 mV/s. The results illustrated the reproducible reaction with both anodic and cathodic peaks, observed at potentials 0.25 and −0.11 V. Additionally, by increasing the number of cycles, the continuous growth of Nq film was obtained. The peak separation potential ΔE_p_ = (E_pa_ − E_pc_) was calculated to be 0.36 V (was higher than 0.059 V) as expected for a quasi-reversible system [48]. Then, the Nq/GCE behavior in 0.10 M PBS (pH 7.0) was examined under various scan rates ranging from 25 to 500 mV/s, as displayed in Figure 6. As can be observed, both anodic and cathodic peak currents gradually increased with a raised scan rate. Furthermore, the oxidative and reductive peak potentials shifted to more positive and negative values. This behavior indicated a surface-controlled process of the electrode reactions process for the organic film of Nq formation [41,49].

### 3.3. Voltammetric Determination of NIC

**Figure 7:**
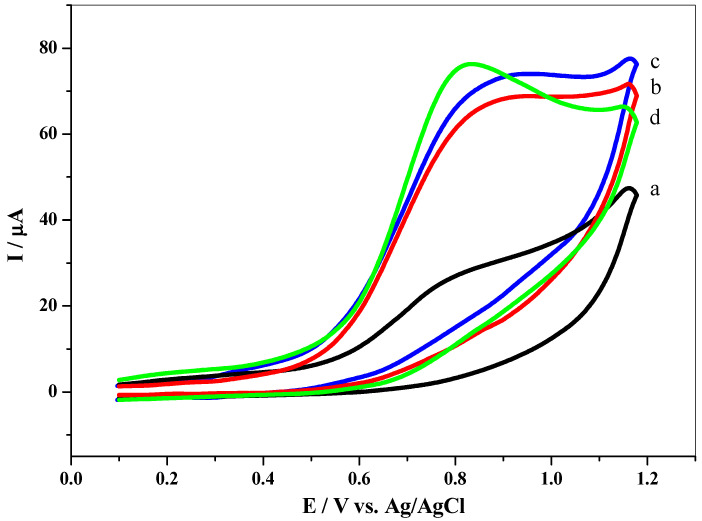
CVs of 3.5 mM of NIC in 0.10 M Na_2_SO_4_(pH 8.0) at (a) a bare GCE, (b) the Nq/GCE, and (d) the GO/GCE (c) the GO/Nq/GCE at a scan rate of 100 mV/s.

Figure 7 describes the CVs of 3.5 mM of NIC in 0.10 M Na_2_SO_4_ (pH 8.0) containing a bare GCE (a), the Nq/GCE (b), the GO/Nq/GCE (c), and the GO/GCE (d) at a scan rate of 100 mV/s. The results showed that the bare GCE (Figure 7, curve a) gave a poorly broad oxidation peak near 0.77 V (vs. Ag/AgCl) is ascribed to NIC oxidation. Meanwhile, in the case of Nq/GCE, GO/GCE, and GO/Nq/GCE, the peak potential of NIC oxidation appeared to be 0.85. 084 and 0.83 V, respectively, as exhibited in Figure 7 (curves b and c). The interpretation of the negative shift in the peak potential is due to the presence of both GO nanosheets and Nq films, which acted as electrocatalysts toward NIC oxidation, which accelerated the electron transfer rate. On the other hand, the usage of GO/Nq/GCE led to the enhancement and increase in the oxidation peak current of NIC by four-fold, compared to that which appeared at bar GCE. This is because the unique conjugation between the Nq film and nanosheets of GO increased the electroactive surface area that catalyzed NIC oxidation [26,27,28]. The absence of voltammetric response (peak) in the cathodic scan is attributed to the irreversibility of NIC oxidation as supported by the literature [24,50].

### 3.4. Effect of pH

**Figure 8:**
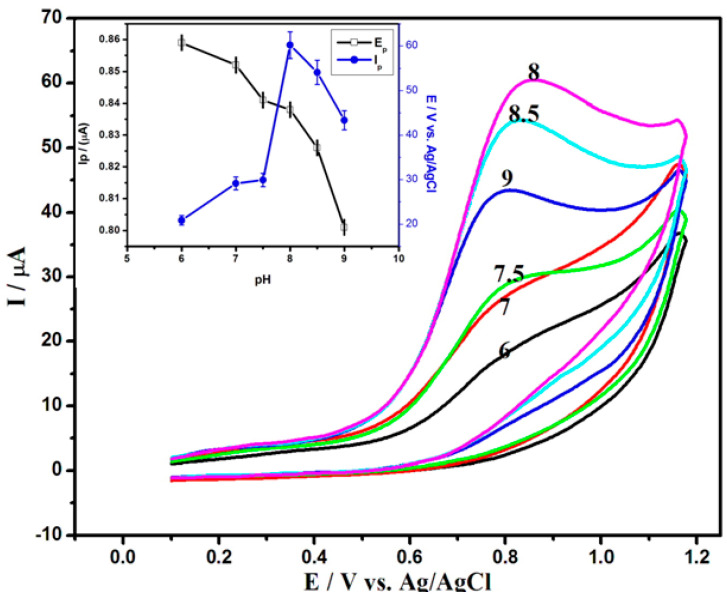
CVs of 3.5 mM of NIC in 0.1 M of Na_2_SO_4_ of various pH values: (6.0, 7.0, 7.5, 8.0, 8.5, and 9.0) at the GO/Nq/GCE with a scan rate of 100 mV/s. (inset Figure 8) A relation between pH versus both oxidation peak potential and oxidation peak current of NIC.

The pH of the supporting electrolyte is considered an essential factor for the peak current (I_p_). By examining the change of pH values from (2.0 to 9.0), it was observed that there are no current responses of NIC at a pH lower than 6.0. However, by increasing the pH value, the anodic peak of NIC increased until a maximum value was observed at pH 8.0, and then it decreased, as shown in Figure 8. This agrees with previous papers that prove that a neutral or slightly alkaline media were appropriate in the voltammetric determination of NIC [5,24]. On the other hand, the potential shifted towards less positive values by increasing the pH values, demonstrating proton participation in the oxidation process [51]. A plot of the oxidation peak potential vs. pH has three almost linear segments, and one is linear over the pH, ranging from 8.0 to 9.0 (inset Figure 8) and expressed by the following equation:

**E_p_(V) = 1.34 − 0.06 Ph  (R^2^ = 0.999)** (2)

The calculated slope of 60 mV is very close to the theoretical value (59 mV) of the Nernst equation. Therefore, the oxidation process of NIC on the surface of GO/Nq/GCE involves the same number of protons and electrons [52,53,54]. This segment of the curves (∼pH 8.0–9.0) is close to the pK_a1_ due to changes in protonation of the acid–base functions in the pyrrolidine moiety. As you know, NIC has pK_a1_ = 8.02, corresponding to the protonation of pyrrolidine nitrogen, and pK_a2_ = 3.12 of pyridine nitrogen [55]. The NIC oxidation process is pH-dependent, and subsequent measurements were carried out in a buffer with pH 8.0.

### 3.5. Effect of Scan Rate

**Figure 9:**
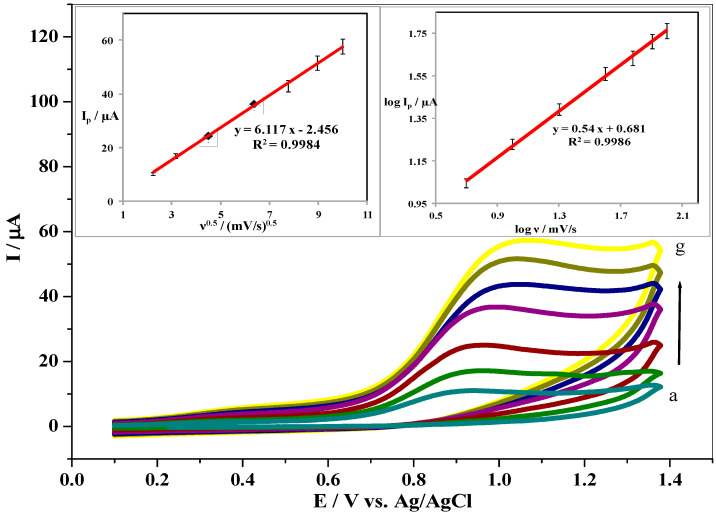
CVs of 3.5 mM of NIC in 0.1 M of Na_2_SO_4_ (pH 8.0) at the GO/Nq/GCE with various scan rates (a to g: 5, 10, 20, 40, 60, 80, and 100 mV/s).

To achieve the optimum conditions, the influence of scan rate on the peak current of 3.5 mM of NIC in 0.10 M Na_2_SO_4_ (pH 8.0)on the surface of the GO/Nq/GCE was investigated. The results demonstrated a linear increase in the peak current of NIC with the increase in scan rate from 5.0 to 100 mV/s, as illustrated in Figure 9. It can be observed that the potential peak shifts toward more positive values with increasing scan rate. Of course, this behavior was characteristic of an irreversible reaction [5,24]. To assess the oxidation process of NIC at the GO/Nq/GCE, a plot of the anodic peak current of NIC vs. square root of the scan rate was recorded (inset Figure 9). A straight linear relationship was obtained, indicating the oxidation reaction of NIC under a diffusion-controlled process and can be expressed as:

**I_p_(µA) = 6.117ʋ^0.5^ − 2.456  (R^2^ = 0.9984)** (3)

For more support, a linear relationship with a slope of 0.54 was obtained when plotting the log peak current versus log scan rate (inset Figure 9) and can be expressed by:

**log I_p_ = 0.54 logʋ + 0.681  (R^2^ = 0.996)** (4)

The slope of the above relation (α = 0.54) is close to the theoretical value calculated, 0.5, as expected for a diffusion-controlled process [56,57,58].

On the other hand, the relation between Ip versus scan rate was studied, a linear relationship was found with correlation co efficient R^2^ = 0.996

**I_p_ = 2.0ʋ − 23.3  (R^2^ = 0.996)** (5)

**Figure 10:**
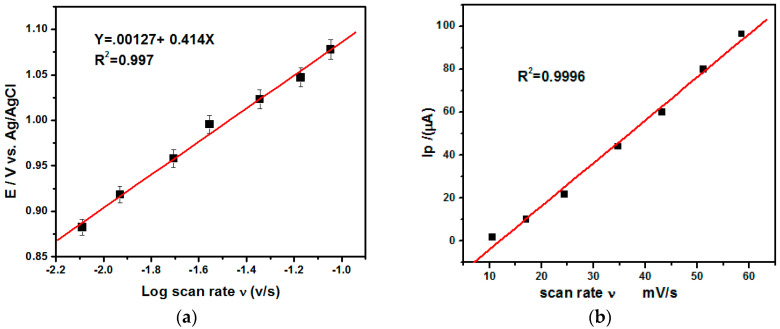
(**a**) The relation between oxidation peak potential of 3.5 mM of NIC in 0.1 M of Na_2_SO_4_ (pH 8.0) at the GO/Nq/GCE versus Log scan rate. (**b**) The relation between peak current of 3.5 mM of NIC in 0.1 M of Na_2_SO_4_ (pH 8.0) at the GO/Nq/GCE versus scan rate.

Moreover, Ep and Log scan rate was determined to inform the number of electron that contributed in the oxidation process of NIC on the surface ofGO/Nq/GCE as seen in Figure 10a,b.

According to the following equation, a linear relation was obtained with correlation co-efficient R^2^ = 99.6.

**Ep = 0.00127 + 0.414logʋ (R^2^ = 0.997)** (6)

**Scheme 2:**
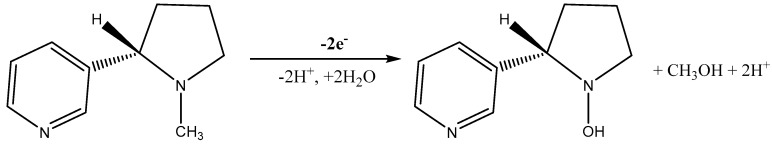
Probable electrochemical oxidation mechanism of NIC.

The number of electrons involved was calculated from the Laviron equation:

**ΔE vs. Δlog(v) = 59/*n*α** (7)

where α is (the electron transfer coefficient) and *n* (refers to number of electrons). The calculated number of electrons were found to be (2.1). That is mean two electron was participated in the oxidation process of NIC. So the expected mechanism (Scheme 2) for the oxidation of NIC at the GO/Nq/GCE in alkaline media which involves substitution of CH_3_ group by OH at the nitrogen of pyrrolidine ring with formation of methanol by two protons and two electrons transfer [5,59].

### 3.6. Effect of Concentration and Calibration Curve

**Figure 11:**
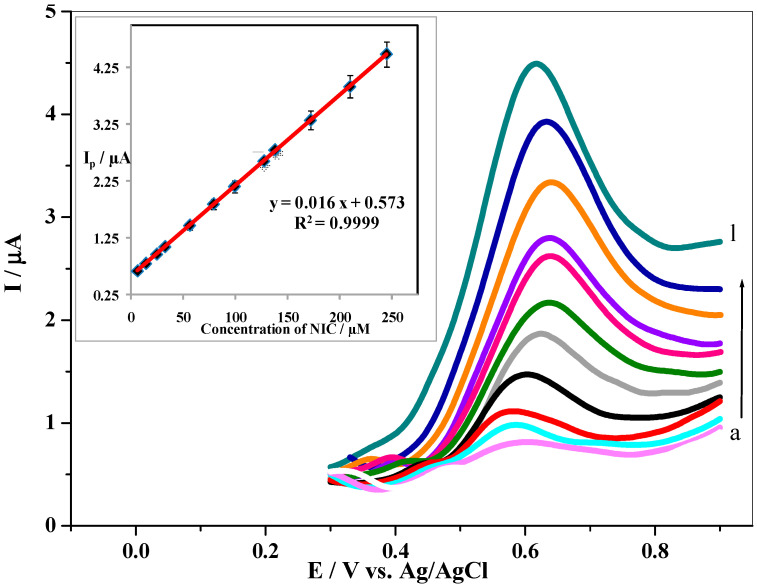
DPVs of 0.1 M of Na_2_SO_4_ (pH 8.0) containing different concentrations of NIC: (a to l: 6.5, 15, 25, 33.5, 56.5, 80, 100, 128, 138.5, 172.5, 210, and 245 µM) at the GO/Nq/GCE. Inset shows the corresponding calibration curve.

The DPV technique is the most appropriate technique used to examine the correlation between the peak current and the standard adding of NIC concentration, characterized by accurate electrochemical determination with a low detection limit and background current [59]. The parameters of DPV were optimized, and the influence of various NIC concentrations at the GO/Nq/GCE in 0.1 M Na_2_SO_4_ (pH = 8.0) was examined. Figure 11 represents the increase in the peak current of NIC by successive standard additions of NIC concentration over the wide range from 6.5 to 245 µM. An excellent linear dependence of the peak current of NIC on its concentration in the same range is shown in Figure 11. The following regression line equation can represent the obtained calibration curve:

**I_p_(µA) = 0.016 (µM) + 0.573  (R^2^ = 0.9999)** (8)

The limit of detection (LOD) and quantification of NIC at the GO/Nq/GCE can be determined according to LOD = 3SD/*b* and LOQ = 10SD/*b*, respectively, where *b* is the slope of the calibration curve. SD is the standard deviation of I_p_ of NIC, estimated from the following equation [60].

**SD = 1/(*n* − 2)Σ(I_exp_ − I_cal_)^2^** (9)

where I_exp_ is the experimental value of I_p_, and I_cal_ is the corresponding calculated value at the same concentration. The calculated LOD and LOQ of NIC at the proposed sensor were 12.7 and 42.4 nM, respectively.

Under the optimum conditions, the sensitivity of our modified sensor was tested. The comparison of the LOD and linearity range obtained in this work with previous reports with different modified electrodes was recorded. The comparison results are epitomized in Table 1. The lower LOD and good linearity range were achieved from the presented data, reflecting the efficiency and sensitivity our modified electrode toward lower concentration of NIC.

### 3.7. Chronoamperometric Measurements

**Figure 12:**
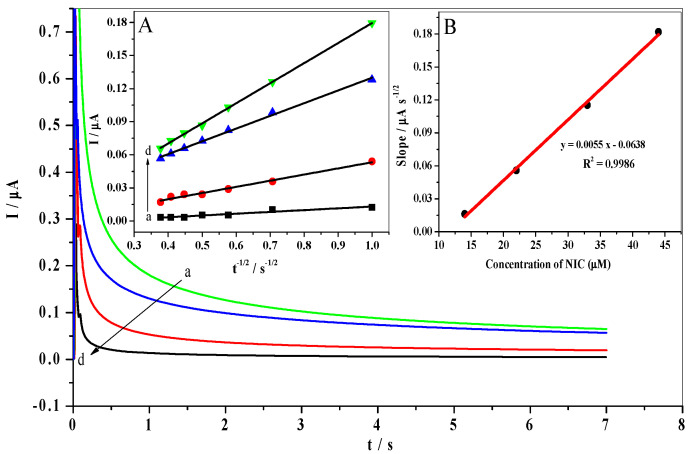
Chronoamperograms were obtained at the GO/Nq/GCE in 0.1 M of Na_2_SO_4_(pH 8.0) for different concentrations of NIC (a to d: 14, 22, 33, and 44 µM). Insets: (**A**) a plot of I vs. t^−1/2^, the (**B**) plot of the slope of straight lines vs. NIC concentration.

The diffusion coefficient of NIC at the GO/Nq/GCE can be determined using the chronoamperometry method. Chronoamperograms were applied by setting the working electrode potential at 0.6 V with various concentrations of NIC (14–44 µM) in 0.10 M Na_2_SO_4_ (pH = 8.0) as represented in Figure 12. Plotting of I for various NIC concentrations against t^−1^ is depicted in Figure 12A. Then, the slopes of the resulting straight lines were plotted versus the different concentrations of NIC that were constructed inset Figure 12B. Using the obtained slope and applying the Cottrell equation [69],

**I = nFAD^1/2^C_b_π^−1/2^t^−1/2^** (10)

where C_b_ (mol cm^−3^) is the bulk concentration, and D (cm^2^ s^−1^) is the diffusion coefficient, the other symbols have their usual meaning. The D value of NIC at the GO/Nq/GCE was determined to be 4.02 × 10^−7^ cm^2^/s is considered a satisfactory result.

### 3.8. Stability and Reproducibility

**Figure 13:**
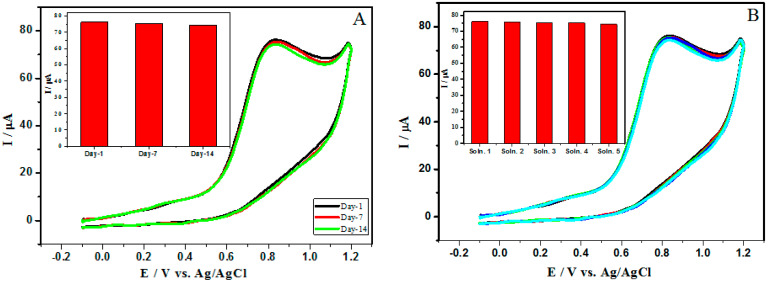
CVs of 3.5 mM of NIC in 0.1 M Na_2_SO_4_ (pH 8.0) at the GO/Nq/GCE with a scan rate of 100 mV/s for 14 days (**A**) and five repetitive CVs measurements (**B**).

The viability of the GO/Nq/GCE could be evaluated by their stability and reproducibility towards the NIC determination. Therefore, Figure 13A shows the CVs of 3.5 mM NIC in 0.10 M Na_2_SO_4_ (pH 8.0) for 14 days, while the modified electrode was kept at 25 °C. The results indicate no significant change in the peak potential, and the peak current remains at 97.71% of its initial current response. Additionally, Figure 13B shows the reproducibility of the GO/Nq/GCE, taking five repetitive CVs’ measurements via the same optimized protocol. It was concluded that the relative standard deviation of RSD = 0.80% in peak current. The results indicated that the fabricated electrode exhibited good stability and high reproducibility.

### 3.9. Interference Study

**Figure 14:**
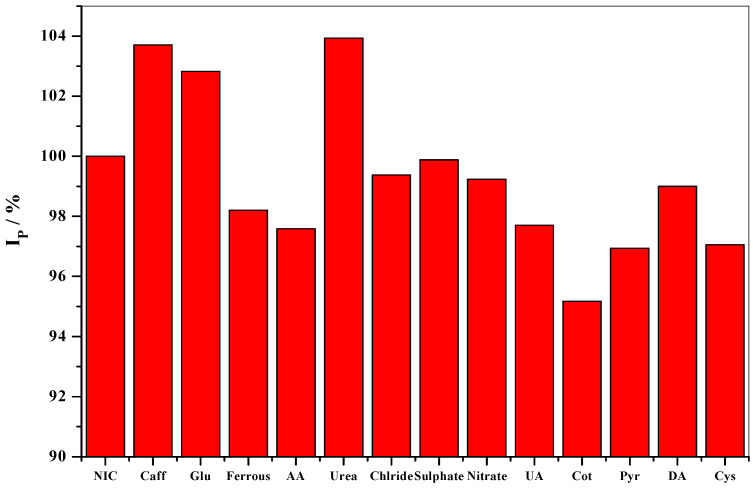
Measurements of 3.5 mM of NIC in 0.10 M Na_2_SO_4_ (pH 8.0) at the GO/Nq/GCE in the presence of the tolerance limit of foreign species.

An important parameter for a developed electrode is its selectivity towards the target analyte. The influence of some possible interferences on the determination of 3.5 mM NIC in 0.1 M Na_2_SO_4_ (pH 8.0) at the GO/Nq/GCE was examined. The tolerance limit was calculated as less than 5% of the relative error in this study. Figure 14 represents the results investigated from the current changes comparing the peak current of NIC only and the signal of NIC with the interfering substances. It is clear that 1000-fold concentration ratios of Na^+^, K^+^, Fe^2+^, Cl^−^, NO_3_^−^, and SO_4_^2−^, 100-fold concentration ratios of glucose and L-cysteine, and 5-fold concentration ratios of pyridine, dopamine, ascorbic acid, and uric acid did not interfere with the measurement of NIC. Additionally, there is no significant variation in the current response after adding 50-fold concentration ratios of urea, caffeine, and cotinine, a primary metabolite of NIC. These results prove the excellent selectivity of the developed sensor for the electrochemical determination of NIC in presences of other interferences.

### 3.10. Real Sample Analysis

**Figure 15:**
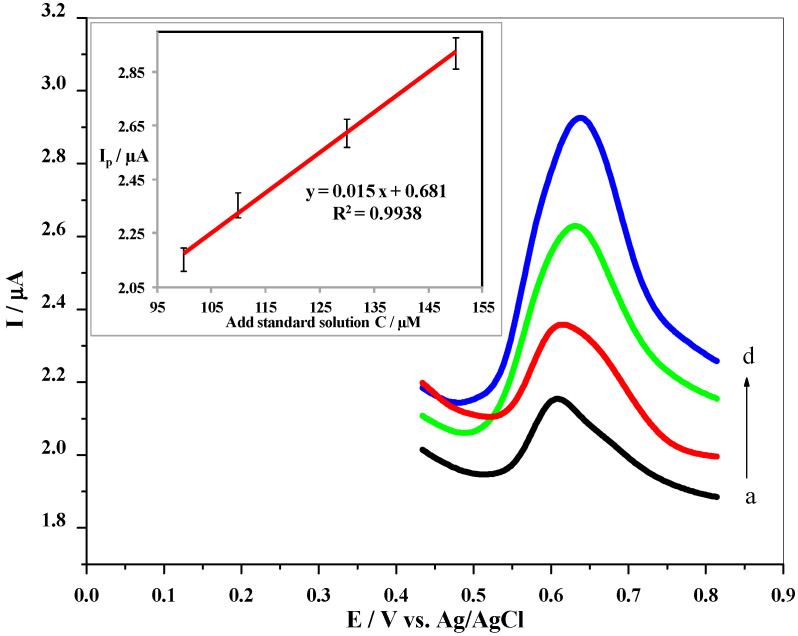
DPVs were recorded at the GO/Nq/GCE upon the addition of known concentrations of NIC (a to d: 100, 110, 130, and 150 µM) in 0.1 M of Na_2_SO_4_ (pH 8.0) with a scan rate of 100 mV/s. Inset shows the corresponding calibration curve.

Next, the content of NIC in a tobacco product and a urine sample was estimated using the GO/Nq/GCE under optimized DPV. Figure 15 depicts DPVs recorded for different concentrations of NIC standard solution, which was added to actual samples in 0.10 M Na_2_SO_4_. At the same time, the final NIC concentrations of 100, 110, 130, and 150 μM and the obtained results are shown in Table 2. As observed, good recoveries were obtained in the range of 99.44–100.33%. Therefore, the proposed sensor is capable of detecting NIC in actual and real samples.

## 4. Conclusions

In this work, we provide an electrochemical sensor used for the first time for NIC determination. The behavior of NIC was studied under optimum conditions. Using different voltammetric techniques such as Cyclic voltammetry. Differential pulse voltammetry and chronoamperometry. The sensor was built on GCE, modified by a layer film of Nq decorated by GO nanosheets. The GO nanosheets were prepared by the Hummers’ method and characterized by different techniques. The fabricated electrode provided a remarkable electrochemical activity toward the oxidation of NIC compared with either bar GCE and GCE modified by Nq. The unique conjugation between Nq and nanosheets of GO has significantly increased the electroactive surface area of the electrode toward NIC.

Additionally, the GO/Nq/GCE showed high stability, reproducibility, a lower detection limit of 12.7 nM, and a linear response range from 6.5 to 450 μM with R^2^ = 0.9999. The oxidation process of NIC is described as control diffusion. The reliable GO/Nq/GCE sensor was successfully applied for detecting NIC in the tobacco product and a urine sample.

## Figures and Tables

**Table 1:** Comparison between our detection limit with the same techniques using different working electrodes.

Technique	Working Electrode	LOD (M)	Linear Range (µM)	Ref.
DPV	SPCE	0.6 × 10^−6^	1–375	[24]
Amp	CuWO_4_/rGO/Nf/GCE	3.5 × 10^−8^	0.1–0.9	[61]
CV	MWCNTs/CuNPs	6.0 × 10^−9^	1–1000	[62]
DPV	Poly(ARS)-GR/SPCE	4.6 × 10^−6^	30–1000	[63]
DPV	RGO/DPA/PGE	7.60 × 10^−6^	31–1900	[64]
CV	MWCNT-BPPG	1.50 × 10^−6^	10–1000	[65]
SWV	p-AHNSA/GCE	0.90 × 10^−6^	1–200	[66]
SWV	EA/GCE	0.70 × 10^−6^	1–200	[67]
SWV	CNC/SPCE	2.00 × 10^−6^	10–1000	[59]
Amp	PDA-RGO/Au/GCE	1.50 × 10^−8^	0.05–500	[68]
DPV	GO/Nq/GCE	1.27 × 10^−8^	6.5–245	This work

**Table 2:** Detection of NIC in practical samples at the GO/Nq/GCE.

Sample	Added (µM)	Founded (µM)	Recovery (%)
Cigarette	0.0	21.13	–
	50	70.73	99.44
	100	121.53	100.33
Urine	50	50.07	100.14
	100	99.63	99.63
	200	199.4	99.7

## Data Availability

Not applicable.
